# Sickle cell disease among Latinx in California

**DOI:** 10.1371/journal.pone.0276653

**Published:** 2022-10-27

**Authors:** Jhaqueline Valle, Judith R. Baker, Daniel Madrigal, Juana Ferrerosa, Susan Paulukonis

**Affiliations:** 1 Tracking California, Public Health Institute, Richmond, CA, United States of America; 2 Center for Inherited Blood Disorders, Orange, CA, United States of America; 3 National Association of Hispanic Nurses, Charles R. Drew University, Los Angeles, CA, United States of America; Loyola University Chicago, UNITED STATES

## Abstract

**Introduction:**

After African Americans, Latinx are the second largest population affected by Sickle Cell Disease (SCD) in the U.S. However, research has largely ignored how this devastating rare blood disorder specifically affects Latinx nationwide.

**Methods:**

This study compared demographics, genotypes, primary insurance, and health care utilization among Latinx and non-Latinx Californians living with SCD, using data from the California SCD Data Collection Program (2016–2018) and newborn screening cases 2000–2017.

**Results:**

Stemming from 6,837 SCD patients, 501(7%) were Latinx. Latinx with SCD (Lx-SCD) were statistically significantly younger than non-Latinx (NLx-SCD) counterparts. Within both groups, females predominated, with 70% being insured by Medicaid. Mean Emergency Department encounters were statistically significantly lower among Lx-SCD adults.

**Discussion:**

Lx-SCD differ in age, genotype, and Emergency Department utilization, when compared to NLx-SCD counterparts in California. Latinx are now the largest racial and/or ethnic group in the US, and their presence in SCD population is expected to grow. Therefore, their specific demographic, genotypic, and health care utilization characteristics merit attention to inform policies and programs that will improve their health.

## Introduction

Sickle cell disease (SCD) is a rare, incurable, and devastating genetic blood disorder whose physical, social, and emotional complications, mortality and costs are elevated [1,2]. The multiplicity of poor health outcomes and costs associated with SCD in the US are aggravated by social determinants of health, since SCD primarily affects people of African descent, burdened with one of the highest national poverty rates [3]. Hispanics/Latinx are at increased risk for SCD [4] and are the second most-prevalent racial/ethnic group carrying SCD [5], though their risk for this disease and its complications is less understood. Higher rates of poverty among Latinx also increases their vulnerability for poor health outcomes within the US. Latinx, now the nation’s largest racial / ethnic minority, also suffer from high poverty rates and related social inequities. Individuals who identify themselves as having more than a single race/ethnicity are growing [6,7]. The increase in US-residing Latinx and mixed-race American populations with SCD is likely to increase. However, few US-based studies have examined SCD within Latinx populations.

SCD affects the shape and function of red blood cells. Although it is rare by US classification, SCD is the most common genetic disease within the USA, affecting approximately 100,000 individuals: 1 in every 365 African American births, and 1 in every 16,300 Hispanic births [4]. SCD complications in children include anemia, stroke, acute chest syndrome, excruciating pain, septicemia, or other serious infections. Debilitating acute and chronic pain episodes are SCD hallmark clinical manifestations within adults, together with strokes, organ damage, and premature death [1,2,8,9]. The course of SCD is partly distinguished by type: hemoglobin Hb S/S and Hb S/β0 are typically more severe, though novel SCD therapies are extending patient lifespan [10], while Hb S/C, Hb S/β+ and other Hb S sub-types typically are milder [2].

Ethnicity is a complex lens through which to view disease. Terminology to describe ethnicity in the US varies over time [6] and is not clearly defined, accurate, or inclusive—and not always accepted [11,12] among the described population. While ‘Hispanic or Latino’ are used by the Census Bureau, this study employed the term Latinx (the non-binary gender form of Latino), acknowledging the complexities of personal ethnic identity.

Screening for SCD among newborns is mandated across all 50 states since 2006, though SCD is not a reportable illness, consequently limiting the understanding of SCD population demographics at both state- and nation-wide levels. One Centers for Disease Control and Prevention (CDC) multi-state surveillance effort found that the incidence of sickle cell trait (carrier) status among newborns by ethnic groups varied widely across states [13]. Similarly, previously published analyses for SCD incidence by ethnicity at state-level are lacking, though the variation in degree of Latinx births among three states (Michigan, 1%; California 7–13%, depending on variant; New York, 11%) suggested that these rates vary widely [14–19]. Information regarding the Latinx SCD population at the state-level–patient demographics, genotype, access to quality healthcare, and utilization of services is a key step to understand and address the impact of this disease–particularly for states where the largest Latinx communities reside, in order to take the necessary steps to improve outcomes.

California’s Sickle Cell Data Collection Program (SCDC) has developed and improved methods and data sources since its inception in 2015. SCDC conducts statewide, longitudinal surveillance of Californians, across all age groups, diagnosed with SCD using linked data from multiple sources. This study aimed to use SCDC data to enhance characterization of demographics, genotype, and health care utilization of California’s Latinx population with SCD on a state-wide level, in order to inform policies and programs that will improve Latinx overall health status.

## Methods

The CA SCDC Program and this study were reviewed and approved by the California Committee for the Protection of Human Subjects, the Public Health Institute IRB, and the IRBs at clinical sites reporting case data. CA SCDC received a waiver of consent.

### Two patient cohorts: Administrative cohort and newborn screening cohort

The SCDC uses population surveillance methods to increase understanding on the care / health outcomes of states’ individual populations living with SCD. Regarding the Administrative Cohort—SCDC collects data and uses patient identifiers to link across diverse sources to create a longitudinal profile. Data sources include Medicaid and Children’s Health Insurance Plan claims for enrollees who have one or more SCD ICD-CM 9 or ICD-CM 10 codes, hospital discharge data, emergency department (ED) encounters, ambulatory surgery encounters, vital records mortality data, newborn screening case reports for those with SCD, and clinical case reports from SCD clinical care centers. Such datasets are linked and de-duplicated on an individual basis using identifiers. A validated case definition for SCD is applied to identify a state-wide cohort, spanning 2005–2018 [20]. These methods are described in more detail within previous literature [21–23].

Individuals in the linked dataset are considered ‘probable’ cases of SCD if identified as having three (or more) separate health care encounters over a five-year period with SCD ICD-9/10-CM. Individuals may also be ‘confirmed’ cases of SCD through a clinical or newborn screening case report, without meeting the above criteria [20]. Individuals included in these analyses met one of these case definitions.

Two subsets of this group were used for sub-analyses–the administrative cohort and the newborn screening cohort. These cohorts are obtained from different datasets, described below, and the best time frame for each cohort was utilized based on the availability of complete data.

**Fig 1:**
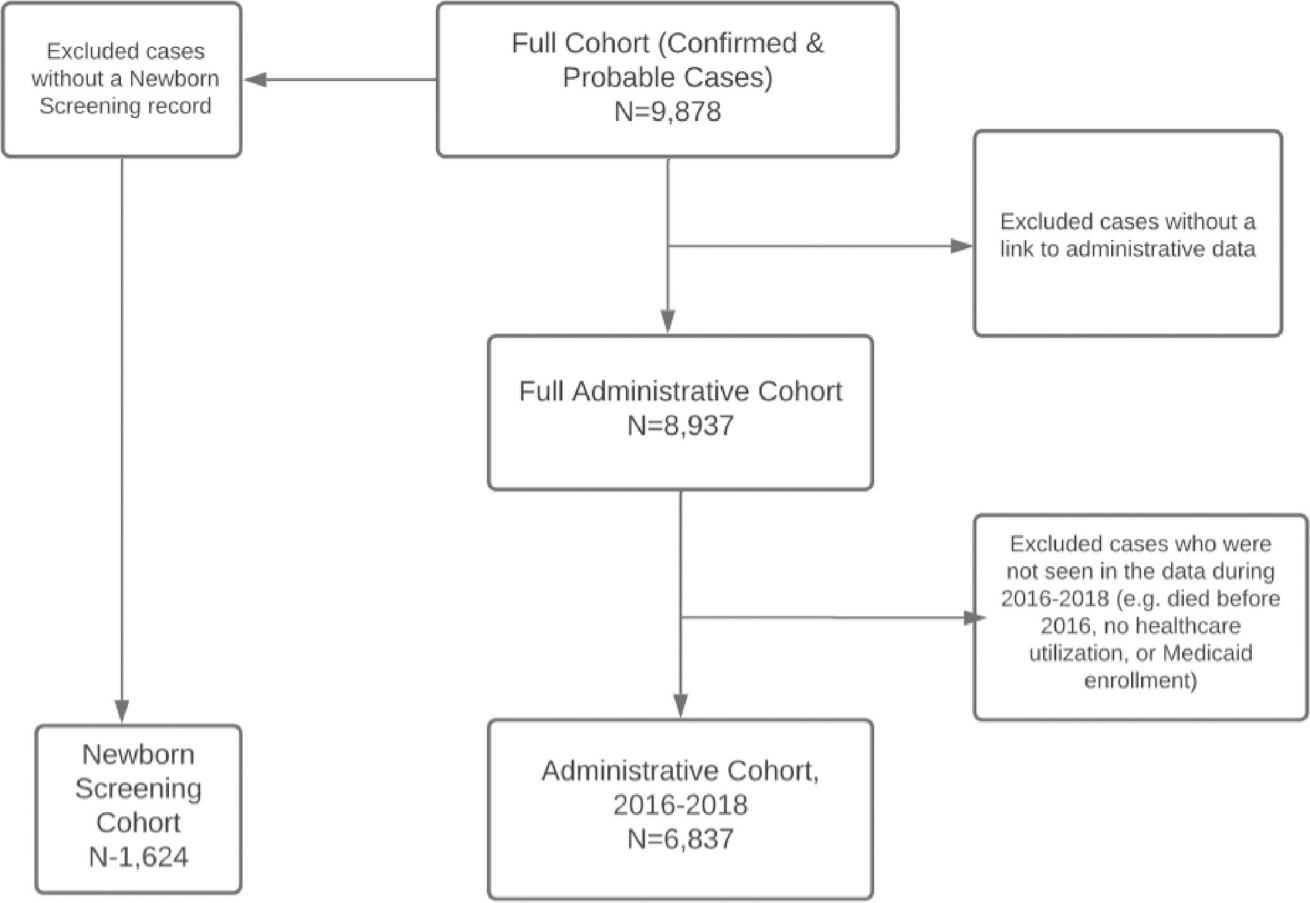
Study subject selection.

The Administrative cohort for this analysis included only individuals residing in California with SCD, who were seen in any of the SCDC datasets (e.g., eligibility files, claims data, hospitalizations, clinic visits etc.) during 2016–2018. Among California’s 8,937 individuals, the entire administrative cohort, comprising 6,837 individuals, met inclusion criteria and were enrolled in this study (Fig 1).

The Newborn screening cohort consists of all newborn screening cases diagnosed with SCD that were reported by the California Newborn Screening Program, between 2000–2017, regardless of linkage to administrative data sources. Concerning this analysis, 1,642 newborns met such inclusion criteria.

### Patient level demographic measures

Demographic measures analyzed in this study were ethnicity, age, geography, gender, access to care, payer, and genotype. Ethnicity was defined as Latinx or non-Latinx, regardless of race, based on data from the state Newborn Screening Program (collected at birth) if available, from clinical case reports, or the most frequently reported ethnicity within administrative datasets (if neither a newborn screening nor clinical report existed for such a case). Unknown or missing ethnicity was considered as non-Latinx. Age groups were defined based on the age of the person at the close of the time period of analysis. Pediatric was defined as less than 21 years of age. Zip-code of residence was based on the last residential zip-code recorded for the individual with SCD within any administrative datasets. California’s Department of Health Care Services certifies facilities [24] as SCD Special Care Centers for the pediatric population. Access to care for children with SCD was characterized by the mean distance from residence to a SCD center, calculated as the distance from the centroid of the most-recent resident zip-code listed, to the zip centroid of the nearest SCD center. Payer type was categorized as Medicaid if the individual was enrolled in Medicaid at last healthcare encounter. If not enrolled in Medicaid, the source of payment, listed in their last encounter, was assigned. Payer categories included Medicare, Private, Self-Pay and Other. Other could include other government programs such as county indigent programs, Title V, and VA. Genotypes were obtained from newborn screening records.

### Health care utilization

The number of non-admitted emergency department (ED) encounters and hospitalizations were drawn from the emergency department and hospitalizations datasets for the period spanning 2016–2018. Mean ED encounters and hospitalizations were calculated and stratified by ethnicity and age group. All encounters, regardless of presenting or discharging diagnoses, were added.

### Statistical analyses

χ2 tests were used to compare categorical variables, t-tests were used for continuous variables. All analyses were performed using SAS 9.4 (Cary™, NC).

## Results

### Demographics

#### Sex/Gender

Among California’s 6,837 residents with SCD in the administrative cohort during 2016–2018, 501 (7.3%) were Latinx. Table 1 depicts demographic characteristics at the end of 2018. Females predominated with 57% and 58% among both Latinx and non-Latinx with SCD, respectively.

**Table 1:** Demographic characteristics of Californians with sickle cell disease by ethnicity, 2016–2018.

	Patients (n = 6,837)		
	Latinx	non-Latinx	P-value
	501(7.3%)	6336 (92.7%)	
Sex (column %)			NS
Male	216 (43.2%)	2668 (42.1%)	
Female	285 (56.8%)	3668 (57.9%)	
Mean (SD) age at the close of 2018, years			
Total	26 (17.7)	33 (18.6)	<0.001
Male	22 (15.7)	30 (18.1)	<0.001
Female	29 (18.6)	35 (18.7)	<0.001
Age at the close of 2018, years (column, %)			<0.001
Pediatric (<20 years)	236 (47.1%)	1721 (27.2%)	
Adult (≥21 years)	265 (52.9%)	4615 (72.8%)	
Age Group (column, %)			<0.001
≤ 9	75 (15.0%)	709 (11.2%)	
10–19	148 (29.5%)	933 (14.7%)	
20–29	114 (22.8%)	1302 (20.6%)	
30–39	64 (12.8%)	1269 (20.0%)	
40–49	35 (7.0%)	836 (13.2%)	
50–59	32 (6.4%)	712 (11.2%)	
60+	33 (6.6%)	575 (9.1%)	
Payer at last encounter (column %)			NS
Medicaid	359 (72%)	4381 (69%)	
Medicare	10 (2%)	240 (4%)	
Private	103 (21%)	1190 (19%)	
Self/Other	29 (6%)	525 (8%)	
Region ^a^ (column %)			<0.05
Los Angeles/Orange County	222 (44.3%)	2462 (38.9%)	
Central Valley	80 (16.0%)	1243 (19.6%)	
Inland Empire	80 (16.0%)	949 (15.0%)	
Greater Bay Area	71 (14.2%)	1051 (17%)	
Imperial/San Diego County	26 (5.2%)	371 (5.9%)	
Other	17 (3.4%)	100 (1.6%)	
Unknown	<1%	<1%	

^a^ Bay Area: Alameda, Contra Costa, Marin, Mendocino, Napa, Santa Clara, San Francisco, San Mateo, Sonoma.

^a^ Central Valley: Butte, Colusa, Fresno, Glenn, Kern, Kings, Madera, Merced, Placer, Sacramento, San Joaquin, Shasta, Solano, Stanislaus, Tehama, Tulare, Yolo, Yuba.

^a^ Inland Empire: Riverside, San Bernardino.

^a^ Other: Humboldt, Modoc, Monterey, San Benito, Santa Barbara, Ventura.

#### Age

Latinxs with SCD (mean age 26 years, median age 22 years) were statistically significantly younger than non-Latinxs with SCD (mean age 33 years, median age 31 years). In addition, 47% of Latinxs with SCD were classified as pediatric, compared to a quarter (27%) of non-Latinx counterparts. This follows equivalent age distributions seen in the general CA population where Latinxs are typically younger than Californians of other races and ethnicities [25]. Notably, 30% of the Latinx population with SCD were in the 10–19-year-old age range, double the proportion compared to non-Latinx with SCD. Males with SCD were typically younger than females with SCD, regardless of race or ethnicity. There has been a slight increase over time in the proportion of Latinx pediatric SCD patients in this state, from 10% in 2008 to 12% in 2018. There was no change evident among the proportion of adult Latinx SCD patients, from 5% in 2008 to 5% in 2018.

#### Geography

SCD cases were clustered in distinct parts of the state, reflecting differences in: A) distribution of overall population: two-thirds of the State’s entire population lives in one-third of the State’s area—Southern CA, Los Angeles/Orange County (LA/OC) in particular, and B) specific geographic areas where Californians of African American and Latinx ethnicity reside. Most Latinx Californians with SCD reside within the LA/OC region followed by the ‘Inland Empire’ and the Central Valley. Non-Latinx Californians also predominantly reside in the LA/OC area, followed by the Central Valley, and the Greater San Francisco Bay Area. Among the pediatric Latinx cohort, the mean distance from a State-designated SCD center was 20.5 miles, as compared to 17.1 miles for non-Latinx, a statistically significant difference.

#### Genotypes

Highlighted in Table 2, among babies born in California between 2000–2017, 11% with SCD were Latinx. SCD genotypes HB SS & SB0, the most severe forms of the disorder, were the most prominent variants, comprising 60% and 55% of Latinx newborns and non-Latinx newborns with SCD, respectively. HB SC, a milder SCD variant, was half as likely to be found among Latinx newborns (14%), compared to 29% of non-Latinx newborns. Conversely, ‘other SCD genotype variants’ were predominantly found in Latinx births (26%), compared to non-Latinx newborns (16%).

**Table 2:** California newborns with SCD 2000–2017 by genotype & ethnicity.

	Total (n = 1,624)		
	Latinx	non-Latinx	P-value
	176 (10.8%)	1448 (89.2%)	
Genotype			
Hgb SS or SB0	106 (60.2%)	790 (54.5%)	NS
Hgb SC	24 (13.6%)	427 (29.4%)	< .0001
Other Variants	46 (26.1%)	231 (15.9%)	< .05

#### Insurance

Medicaid was the payer of record for > 70% of patients (72% Latinx: 69% non-Latinx) during the period of analysis.

#### ED visits and hospitalizations

Nearly two-thirds of all Latinx patients and 73% of non-Latinx had one (or more) non-admitted ED visit (for any diagnosis) during the 2016–2018 period; 46% and 54%, respectively, had one (or more) hospitalizations (Table 3). Patterns differed by age. Children with SCD, regardless of race and ethnicity, had similar numbers of mean ED visits / hospitalizations over the 2016–2018 period. Patterns of hospitalizations among adults were also similar, regardless of race and ethnicity. However, mean (though not median) ED encounters were lower among Latinx adults with SCD (5.3), compared to non-Latinx counterparts (8.2).

**Table 3:** Hospitalizations and emergency department encounters, all-cause, among Californians with SCD, Latinx versus non-Latinx, 2016–2018.

	Pediatric			Adult			Total		
	Latinx	non-Latinx	P-value	Latinx	non-Latinx	P-value	Latinx	non-Latinx	P-value
**Hospitalizations**									
**Total Hospitalizations**	377	2,494		1,058	17,216		1,435	19,710	
Mean Hospitalizations	1.6	1.5	NS	4	3.7	NS	2.9	3.1	<0.05
Median (min-max)	1 (0–29)	1 (0–72)		2 (0–71)	3 (0–433)		1 (0–71)	2 (0–433)	
1 or more	76 (32.2%)	617 (35.8%)	NS	154 (58.1%)	2881 (62.4%)	NS	230 (45.9%)	3428 (54.1%)	< .05
**ED Encounters**									
**Total ED Encounters**	565	4,007		1,401	37,850		1,966	41,857	
Mean ED Encounters	2.4	2.3	NS	5.3	8.2	< .05	3.9	6.6	< .001
Median (min-max)	1 (0–29)	1 (0–72)		2 (0–71)	3 (0–433)		1 (0–71)	2 (0–433)	
1 or more	125 (53.0%)	869 (50.5%)	NS	204 (77.0%)	3733 (81.8%)	NS	329 (65.7%)	4602 (72.6%)	< .05

## Discussion

To our knowledge, this is the largest longitudinal state-wide surveillance effort that specifically compared demographics, genotype, insurance, and health care utilization between Latinx and non-Latinx individuals living with SCD. Such data illustrated that in California, a substantive and growing portion of the population living with SCD is Latinx. Furthermore, such data indicated variations in age, genotype, and healthcare utilization among Latinx Californians with SCD, as compared to non-Latinx counterparts. Both racial and ethnic groups shared a predominant reliance on Medicaid. These patterns have implications for program and policy planning, healthcare delivery, workforce development, cultural competency, and adolescent transition.

Moreover, since Latinx now comprises the largest US racial / ethnic minority, such findings are nationally relevant. Across the country, sizeable state-, county- and city-specific Latinx populations are geographically distant from one another. This renders a regional approach to SCD services–first introduced by the US Health Resources and Services Administration’s (HRSA) SCD Treatment Demonstration Program in 2014 –vital to rapidly disseminate and accelerate adoption of promising practices to best serve Latinx with SCD [26–28].

The larger-portion Latinx with SCD among the pediatric population, compared to non-Latinx counterparts, has program planning and policy implications. As state and national demographics shift to higher proportions of Latinx and individuals of mixed race (and corresponding lower proportions of African Americans), Latinx SCD populations will eventually also expand. SCD clinics and CBOs (community-based organizations) must collaborate to effectively address school attendance, language, and cultural needs of their Latinx populations, and to appropriately promote transition from pediatric to adult care. Adolescent transition is a priority of many State Title V programs for special-needs children and HRSA’s regional SCD demonstration programs [27].

This study found a higher proportion of adult females in this dataset, when compared to adult males. The variations in gender proportions when comparing the full population of individuals with SCD to the Latinx population are minimal, though it bears noting that these variations in enrollment by gender were observed throughout the Medicaid system among adults. In 2010, California’s Medi-Cal program [29] enrolled 33% male and 67% female among 19–44-year-olds, while in 2020 the split was 43% male and 57% female, as reflected within Table 1. Women were more likely to be sole-providers for children and were eligible while pregnant and in the post-partum period, all being qualifying conditions—along with income.

Several findings merit attention. This cohort’s Latinx newborns with SCD were 50% less likely to have a less severe (Hemoglobin S/C) form of the disease in comparison to non-Latinx (mirroring a New York SCD genetic variation study of newborns to foreign-born Latinx mothers) [18], and more likely to have higher levels of rare SCD variants. Outside of the US, data is sparse on the distribution of genotypes among Latinx populations with SCD [14], with most Latin American countries only reporting the prevalence of sickle cell trait and sickle cell anemia. Within SCD, severity level predicts higher rates of vaso-occlusive crises which are associated with poor patient-reported outcomes, lower income, productivity, and need for disability insurance [30]. Understanding how genotype and phenotype impacts different ethnic groups living with SCD will accelerate the establishing of practices and policies that more precisely address patient requirements, to improve health-related quality-of-life and outcomes.

The geographic distribution of both the Latinx and the non-Latinx populations with SCD is similar in California. Slightly higher proportions of Latinx with SCD live in Los Angeles County; while larger proportions of non-Latinx counterparts reside in the Central Valley and the Greater San Francisco Bay Area. Such patterns align with broader distributions of Latinx and non-Latinx populations without SCD, within the state. These regional clusters can help inform resource decisions regarding SCD related in outreach, and access to knowledgeable adult healthcare providers and Community Health Workers [31].

This cohort’s uniformly high reliance on Medicaid, regardless of race or ethnicity, confirms how critical Medicaid policies are for the SCD population on a state-wide level. Medicaid policies must allow patient access to specialized SC Centers, must optimally reimburse for Community Health Workers and for team-based outpatient clinical care services, and must also automatically qualify individuals with SCD as eligible for complex care services that address medically and economically vulnerable individuals who, by definition, have heightened social, mental, and behavioral health needs.

Such findings that revealed mean annual ED encounters (2016–2018) were statistically significantly lower among Latinx adults with SCD, compared to non-Latinx counterparts, are concerning. Further research is needed to better understand the underlying roles that English proficiency, insurance, immigration status, scarcity of specialized outpatient clinics for adults with SCD, geography, and age-related SCD complications play, and the implications for health outcomes.

Such data illuminate this emerging population to stimulate service enhancements and suggest areas for further research. There have been no other US state-wide population-based investigations of this scope examining the prevalence and characteristics of the Latinx SCD population. This information fills an important gap in the literature.

Concerted efforts are underway to address the needs of California’s Latinx SCD population. The Pacific Sickle Cell Regional Collaborative (PSCRC), established in 2014 as one of five HRSA SCD Treatment Demonstration Project Regions, initiated a partnership with the National Association of Hispanic Nurses-Los Angeles (NAHN-LA) in 2015, focusing firstly on local SCD clinical workforce development and public outreach/awareness, subsequently expanding to initiatives with a national reach [28,32]. California’s 2018 Sickle Cell State Action Plan notes the importance of understanding the experience and outcomes of different racial and ethnic groups with SCD, and includes related recommendations [32]. California Assembly Bill 1105, introduced by Assembly member Mike Gipson (D-Los Angeles) in the 2019–2020 legislative session, was funded as SB 74 in Governor Newsom’s Budget Act of 2020, creating Networking California for Sickle Cell Care (NCSCC) [32] to build a network of adult sickle cell centers, expand workforce, enhance surveillance, and increase education and outreach. NAHN-LA is one recipient of an NCSCC competitive education/outreach grant.

Such data documents an estimated 500 Latinx Californians with SCD—this number is expected to increase with the proactive case finding efforts of a growing cadre of NCSCC-funded SCD community health workers, and as novel NCSCC-supported adult SC Clinics are established state-wide. Healthcare providers, community-based organizations, researchers, and policy makers should understand the needs of Latinx with SCD to devise programs to improve their health outcomes. Increased access to culturally appropriate education materials for Latinx with SCD are needed.

Additional research is required in numerous areas comparing Latinx with SCD to non-Latinx counterparts, that hold promise to improve health outcomes, namely: How does prevalence of genotypes and phenotypes vary and what are the implications for wellness, disease complications, cost, and mortality? How do access to outpatient, inpatient, and ED care vary? How does time from symptom to diagnosis and utilization of preventative screenings and disease-modifying therapies differ? How does stigma manifest and what programs optimally reduce it? What clinical and community health worker training programs show promise in addressing the specific needs of different racial and ethnic populations with SCD? What culturally appropriate modifications to adolescent transition programs show promise for differing SCD populations?

### Limitations and strengths

One limitation of this analysis was the definition of SCD centers used to measure distance travelled to obtain expert care. SCD ‘Special Care’ centers are designated by the State Department of Health Care Services. The approved list primarily consists of pediatric SCD centers; frequency of facility evaluation and how often the list is updated are not clear. Secondly, there is documentation of over two thousand SCD-related healthcare encounters annually, to individuals who lack a social security number (SSN) or other identifying information (unpublished data) that were not explored in this study. Whether these encounters represented true SCD cases, and whether the absence of SSN was due to undocumented status, are unknown. Additionally, because the SCDC surveillance system relies on administrative claim data from Medicaid and Hospital/Emergency departments to identify persons with SCD using a validated case definition [20] based on ICD codes, there is a risk of missing people who are not connected to care, un-insured, privately insured, or not utilizing the healthcare system. Finally, the use of administrative, rather than clinical data, for much of these analyses rendered finer-grained comparisons challenging. Despite these limitations, this state-wide population of Latinx with SCD, derived from a robust multi-source methodology, was the largest reported in the US. Such data are longitudinal, reducing year-to-year fluctuations, and are thus more reliable in understanding trends over time, geography, payer, and health complications, as well as race and ethnicity of cohort constituents [33].

## Conclusion

The numbers of Latinx with SCD is also expected to grow in the US, and this population warrants attention. This largest assessment of Latinx Californians with SCD using state-wide surveillance data, revealed differences in genotype, sex, age, geographic distribution, and healthcare utilization, though also had similarities in high reliance on Medicaid insurance, compared to non-Latinx counterparts. The authors encourage other states to utilize these methodologies to raise awareness, inform policy, and program development to this underserved, medically vulnerable population.
